# Prenatal diagnosis of Roberts syndrome in a Chinese family based on ultrasound findings and whole exome sequencing: a case report

**DOI:** 10.1186/s12920-022-01161-8

**Published:** 2022-01-29

**Authors:** LiFen Zhu, DingYa Cao, Min Chen, Huimin Zhang, XiaoFang Sun, WeiQiang Liu

**Affiliations:** 1 Department of Obstetrics and Gynecology, Key Laboratory for Major Obstetric Diseases of Guangdong Province, The Third Affiliated Hospital of Guangzhou Medical University, Guangzhou, 510150 Guangdong China; 2 Central Laboratory, Longgang District Maternity & Child Healthcare Hospital of Shenzhen City, Shenzhen, 518172 China; 3 Key Laboratory of Reproduction and Genetics of Guangdong Higher Education Institutes, Guangzhou, 510150 China; 4 Department of Obstetrics and Gynecology, Department of Fetal Medicine and Prenatal Diagnosis, Key Laboratory for Major Obstetric Diseases of Guangdong Province, The Third Affiliated Hospital of Guangzhou Medical University, Guangzhou, 510150 China

**Keywords:** Prenatal diagnosis, Roberts syndrome, Ultrasound, Whole exome sequencing, ESCO2

## Abstract

**Background:**

Roberts syndrome (RBS) is a rare autosomal recessive disorder caused by variations in the *ESCO2* gene; however, prenatal diagnosis of RBS has never been reported in Chinese families. Additionally, fetal-specific phenotypic characteristics associated with *ESCO2* variants have not been reported.

**Case presentation:**

A fetus in a healthy, nonconsanguineous Chinese family with multiple serious congenital malformations was diagnosed prenatally. Two consecutive fetuses in this family presented with tetraphocomelia, growth restriction, cleft lip and palate bilaterally, and other abnormalities. The main phenotypic characteristics of this case were strongly suspected to be associated with RBS. Finally, whole exome sequence analysis revealed the insertion of a homozygous base pair in exon 6 of the *ESCO2* gene (NM_001017420.3, c.1111insA, NP_001017420.1, p.Thr371fs). Both of the couples were heterozygous carriers for this variant.

**Conclusion:**

We are the first to report a prenatal case of RBS diagnosed in a Chinese family. Here, we have confirmed that the rare variant is a definite pathogenic variant, and we provide detailed phenotypic characteristics for the prenatal diagnosis of RBS due to this causative variant.

## Background

Roberts syndrome (RBS, OMIM 268300) is a rare autosomal recessive disorder caused by variants in the establishment of the cohesion 1 homolog 2 (*ESCO2*) gene [1]. The main characteristics of RBS are prenatal and postnatal growth retardation, tetraphocomelia and craniofacial abnormalities (ranging from mild to severe). Hand abnormalities, ear abnormalities, and cardiac or kidney abnormalities may occasionally occur in affected individuals. The most serious RBS cases result in spontaneous abortion, stillbirth or death within 1 month [2]. However, some mildly affected patients can survive into adulthood with varying degrees of intellectual disability [3].

The prevalence of RBS is unclear. To date, approximately 150 cases of RBS have been reported in the literature [3]. Although the incidence of RBS is not affected by race, a Chinese case of RBS diagnosed prenatally has not yet been reported in the literature. Here, we present a case of RBS diagnosed prenatally with a severe phenotype in a Chinese family. The clinical diagnosis is based on fetal anomalies detected by ultrasound followed by a molecular diagnosis by whole exome sequencing.

## Case presentation

A 29-year-old pregnant woman (G2P0) and her husband were referred to our fetal medicine department for genetic counseling. On the 22nd week of her first pregnancy, the female fetus had multiple serious congenital anomalies, including limb malformation, bilateral cleft lip and palate, and severe growth restriction. They opted for termination of pregnancy, and no other survey was conducted for the aborted fetus. The healthy, nonconsanguineous couple of Chinese origin denied any other family history of congenital diseases and had no history of exposure to medication during pregnancy.

**Fig. 1:**
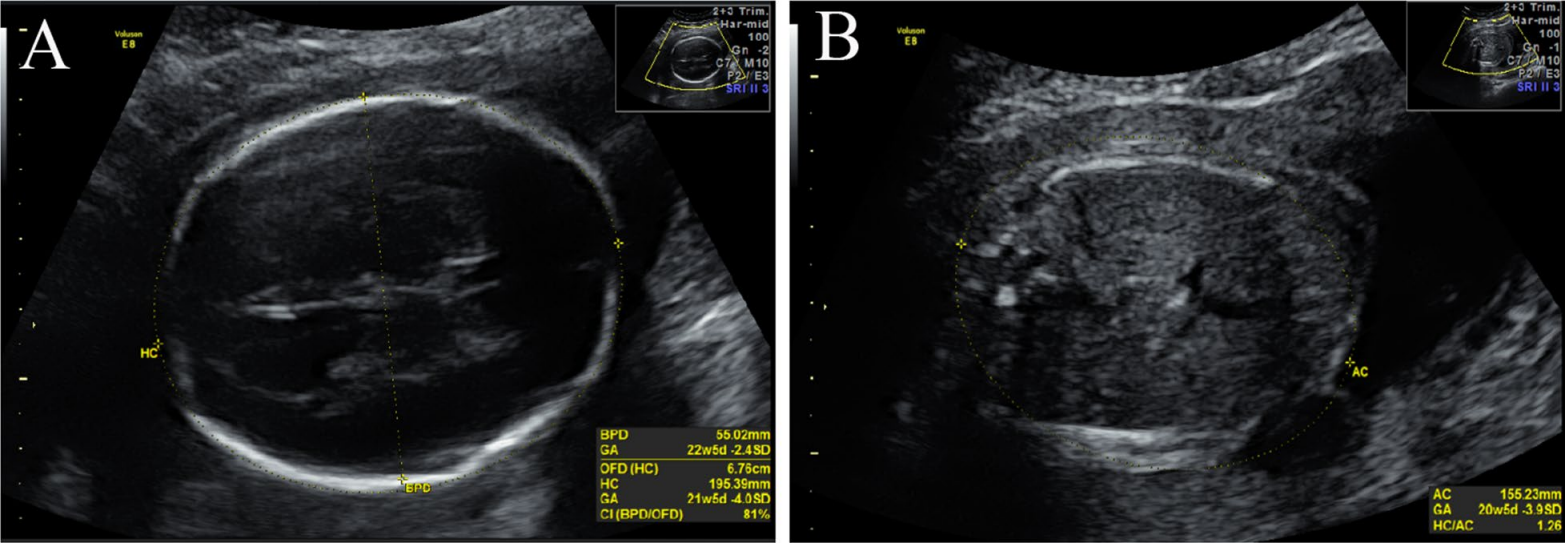
Fetal ultrasound at 25 weeks of gestation indicates severe growth restriction. BPD, HC (**A**) and AC (**B**) measure below the 3rd percentile

During her current pregnancy, the woman did not have regular physical examinations. At 25 weeks of gestation, ultrasound examinations performed at the local hospital again showed multiple similar congenital malformations of the fetus. At the same time, a detailed ultrasonic examination conducted in our hospital showed a singleton male fetus with severe growth restriction, with a biparietal diameter (BPD) of 55.02 mm (− 2.4 SD, the 0.8 percentile), occipito-frontal diameter (OFD) of 67.6 mm (− 4.0 SD, the 0.03 percentile), head circumference (HC) of 195.4 mm (− 4.0 SD, the 0.8 percentile), abdominal circumference (AC) of 155.23 mm (− 3.9 SD, the 0.05 percentile) and estimated fetal weight (EFW) of 378 g (− 1.7 SD, the 4.457 percentile) (Fig. 1).

**Fig. 2:**
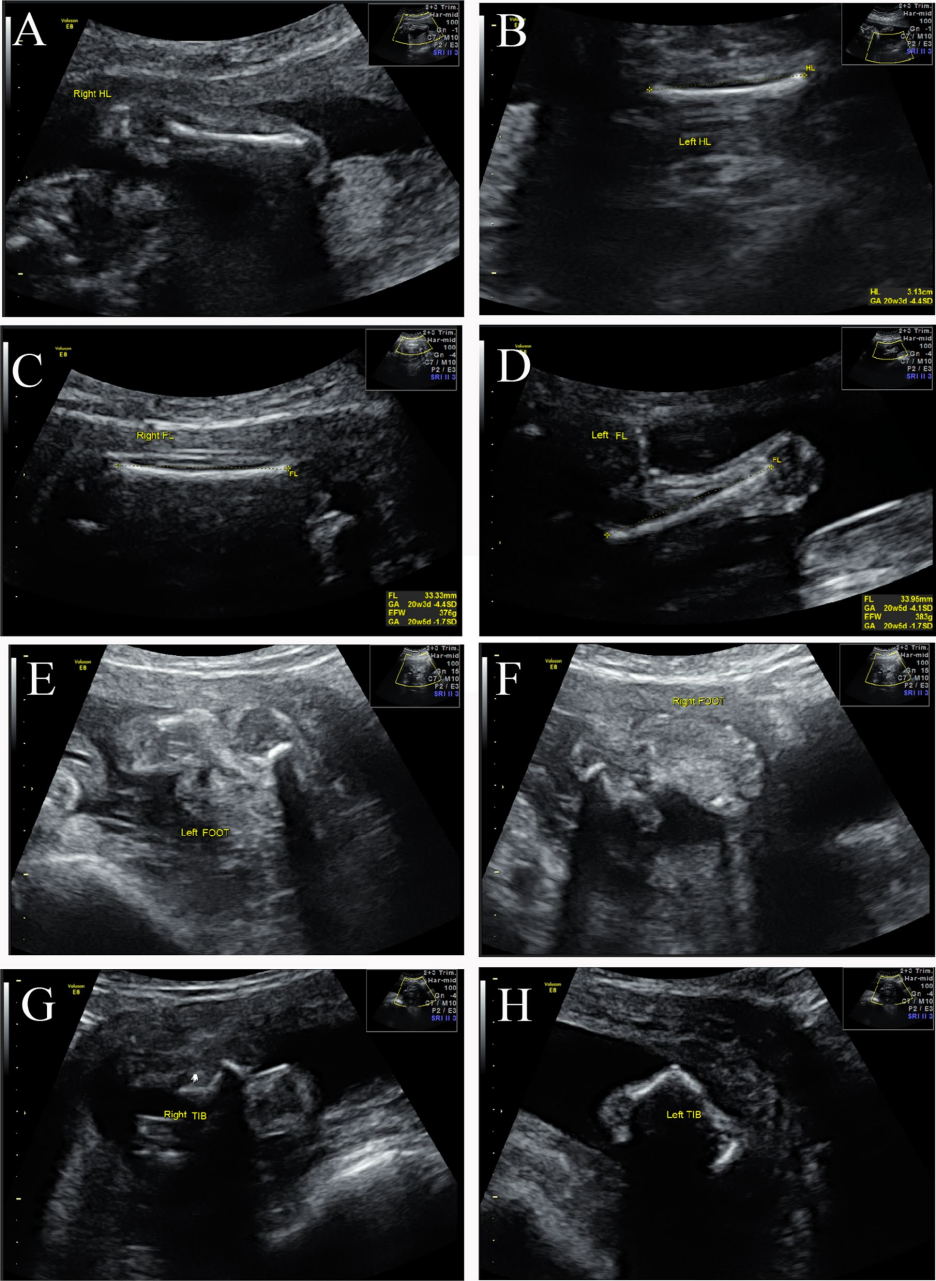
The extremities were significantly reduced bilaterally in the humerus (**A**, **B**) and femur (**C**, **D**). The fetus had flexural contractions in the elbow, knee and ankle on both sides, as well as equinovarus talipes (**E**–**H**)

The fetus had flexion contractions in the elbow, knee and ankle on both sides, as well as with equinovarus talipes. The extremities were significantly shortened bilaterally in the humerus and femur. The length of the right and left humerus was 29.3 mm (− 5.2 SD) and 31.3 mm (− 4.5 SD), while the length of the right and left femurs was 33.3 mm (− 4. 4SD) and 33.9 mm (− 4.1 SD), respectively. The bilateral radius, ulna and fibula were apparently not presented (Fig. 2).

**Fig. 3:**
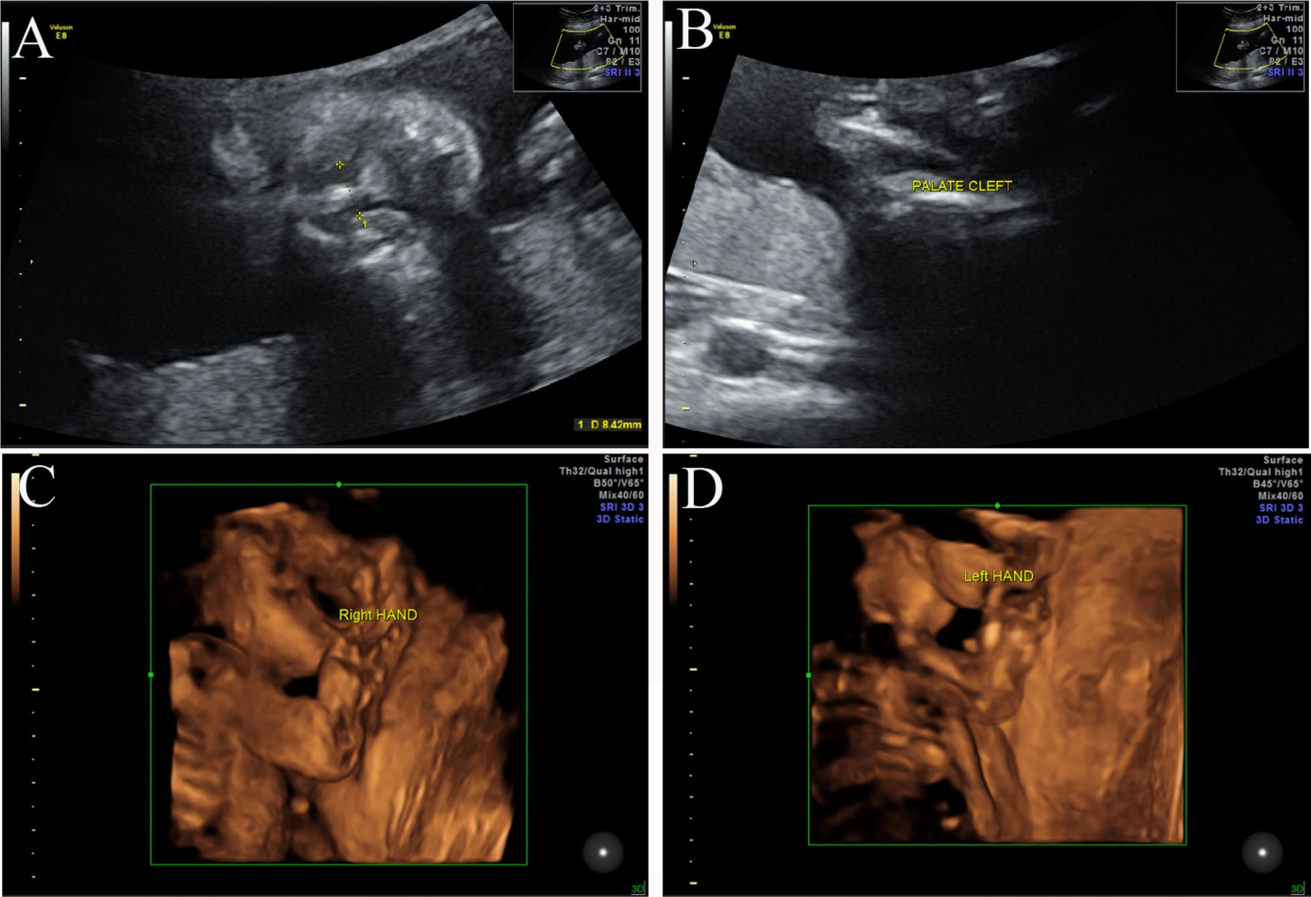
Ultrasound examinations reveal a bilateral cleft lip and cleft palate in the fetus (**A**, **B**), the absence of the right thumb and the presence of an appendicular thumb on the left (**C**, **D**)

Ultrasound examination of fetal hands revealed the absence of the right thumb and the presence of an appendicular thumb on the left. A bilateral cleft lip and cleft palate were also identified (Fig. 3). No internal abnormalities or polyhydramnios were observed, and the genitourinary system was normal.

The couple opted for pregnancy termination and decided to proceed with genetic testing to determine the causative genetic factors. Postmortem examination confirmed the ultrasound findings mentioned above.

**Fig. 4:**
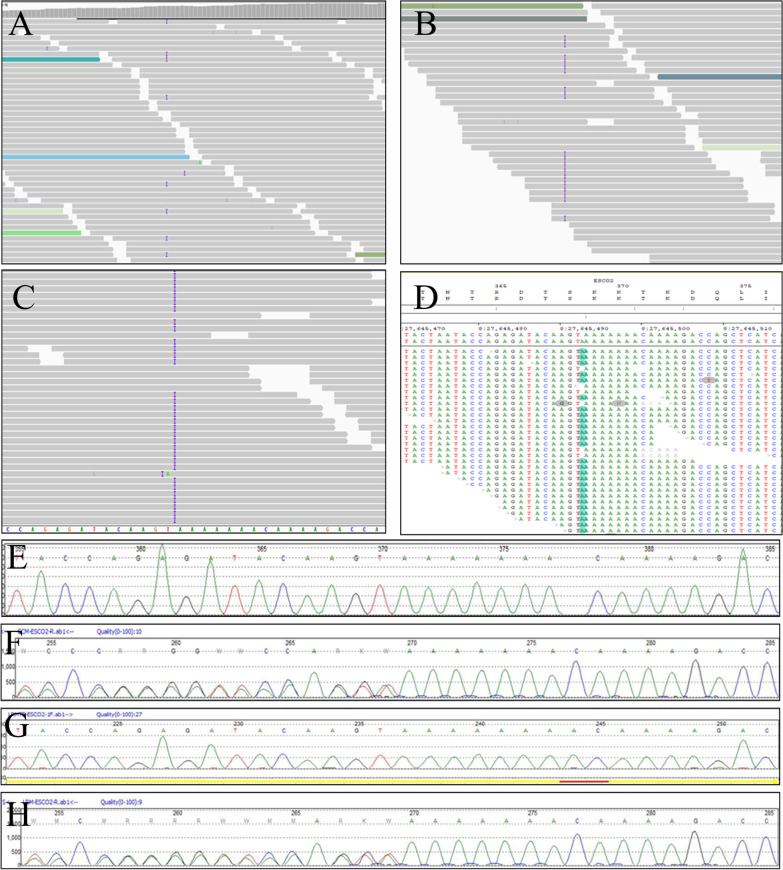
Whole exome sequencing identified a heterozygosity frameshift insertion of 1 base pair involving exon 6 of the *ESCO2* gene (NM_001017420.3, c.1111insA, NP_001017420.1, p.Thr371fs) in the couple (**A**, **B**) and a homozygous mutation of this allele site (**C**, **D**). Sanger sequencing confirmed the mutation. Both members of the couple were heterozygous for the mutation (**F**, **H**), and the fetus had a homozygous mutation (**G**). **E** Represents a reference sequence of the *ESCO2* gene

For genetic assessment, 10 ml of fetal amniotic fluid and 5 ml of parents' peripheral blood were collected for fetal-only chromosomal microarray analysis (Thermo Fisher, Affymatrix Cytoscan 750 K) and trio-whole exome sequencing (Mingma Technologies Co. Ltd) to detect copy number variations and gene variants, respectively. No pathogenic or likely pathogenic copy number variants were identified in the fetus. A trio-whole exome sequencing analysis revealed a homozygous frameshift insertion of a homozygous base pair in exon 6 of the *ESCO2* gene (NM_001017420.3, c. 1111insA, NP_001017420.1, p. Thr371fs), and both couples were heterozygous carriers for this variant. Sanger sequencing was performed and confirmed this variant (Fig. 4).

## Discussion and conclusions

RBS, also known as Roberts-SC phocomelia syndrome or ESCO2 spectrum disorder, is a rare autosomal recessive disorder caused by variants of the *ESCO2* gene [4]. Patients with RBS have a variety of signs, ranging from serious malformation to milder forms [2, 5, 6]. The pathogenesis of RBS is caused by a variant in the *ESCO2* gene located at 8p21.1, which encodes a protein with the function of acetyl-transferase in establishing sister chromatid cohesion during S phase and mitosis [5]. In RBS patients, *ESCO2* gene defects lead to the loss of acetyltransferase activity, sister chromatid polymerization failure and centromeric separation, and they result in very slow or even interrupted cell division and abnormal gene expression patterns [7].

For prenatal diagnosis of RBS, there must be careful differentiation between cases where the conditions are clinically similar. For example, limb anomalies and growth retardation can be observed in RBS, Cornelia de Lange syndrome, CHARGE syndrome, Tetra-melia syndrome 1 and thrombocytopenia-absent radius syndrome. Therefore, identification of the causal genetic factor plays a critical role in the clinical diagnosis or future preimplantation genetic testing of these diseases. In this case, a fetus in a healthy, nonconsanguineous Chinese family with multiple serious congenital malformations was diagnosed prenatally. Two consecutive fetuses in this family had tetraphocomelia, growth restriction, bilateral cleft lip and palate and other anomalies. Most of the clinical parameters observed are below the 3rd percentile. The key phenotypic features of our case are strongly suspected to be associated with RBS.

Although approximately 150 cases of RBS have been reported, the exact prevalence of RBS is unknown. To date, there are approximately 40 pathogenic or likely pathogenic *ESCO2* variants listed in the ClinVar database (https://www.ncbi.nlm.nih.gov/clinvar) or in the literature [8, 9], and most of them are frameshift, nonsense or splice-site variants. Regarding our case, the c.1111dupA variant in exon 6 of the *ESCO2* gene is not present in population databases (such as the ExAC and gnomAD databases). Although this variant has been reported three times in individuals with RBS, for which it has been classified as a pathogenic or likely pathogenic variant (https://www.ncbi.nlm.nih.gov/clinvar/variation/21232/), the fetal-specific phenotypic characteristics correlated with this variant have not been reported.

We reviewed the relevant literature and databases, and unfortunately, the prevalence of the disease and this variant in the Chinese population is not known. Although the couple denied consanguinity, we cannot rule out the possibility of a common ancestry between them, given that both members of the couple are carriers of the same very rare variant.

According to the gene variant interpretation guidelines [10] and the similar classical RBS phenotype observed in the consecutive two fetuses, we classified this variant as a definitive pathogenic variant based on the following criteria: PVS1-VS (pathogenic criterion is weighted as very strong), PP4 (patient’s phenotype or family history is highly specific for a disease with a single genetic etiology), and PM2-supporting (absent from controls or population database if recessive in Exome Sequencing Project, 1000 Genomes Project, or Exome Aggregation Consortium).

Although the c.1111dupA variant has already been described once previously [4], the clinical information, particularly the phenotype present during pregnancy, is not fully provided. Based on detailed prenatal ultrasound results and molecular tests, we demonstrated that the c.1111dupA variant in the *ESCO2* gene could cause a severe and classical phenotype of RBS.

Genetic testing of carrier status for the parents was indicated to calculate the risk of having another child with RBS in a future pregnancy. As the genetic analysis revealed that both parents are carriers of the c.1111dupA variant of the *ESCO2* gene, the parents were informed that they have a 25% risk of having another affected child and were discussing options regarding a future pregnancy. In addition, fetal cells can be obtained by amniocentesis extraction of amniotic fluid, and cytogenetic analysis of fetal DNA, which is the gold standard for the diagnosis of RBS during pregnancy, can confirm the diagnosis [11].

In conclusion, we report, for the first time, a case of prenatally diagnosed RBS in a Chinese family. We have confirmed that the rare variant is a definite pathogenic variant and provided detailed phenotypic characteristics for the prenatal diagnosis of RBS due to this causative variant. Studies on the pathogenesis of such recurrent congenital malformations in whole exome sequencing provide the basis for genetic counseling to avoid the risk of recurrence and complications in future pregnancies.

## Data Availability

All data generated or analyzed in this study are included in the published article. whole exome sequencing data for fetuses and parents of Variable Sites are available in the NCBI SRA under the accession numbers PRJNA788695 (https://www.ncbi.nlm.nih.gov/Traces/study/?acc=PRJNA788695).

## Abbreviations

RBS: Roberts syndrome
BPD: Biparietal diameter
SD: Standard deviation
OFD: Occipito-frontal diameter
HC: Head circumference
AC: Abdominal circumference
EFW: Estimated fetal weight
